# Community pharmacy density at national and subnational levels in New Zealand

**DOI:** 10.1080/03036758.2025.2488394

**Published:** 2025-04-08

**Authors:** Michael J. Leach

**Affiliations:** School of Rural Health, Monash University, Bendigo, Australia

**Keywords:** Community pharmacy, medicines, accessibility, geographic information systems, health services research, policy

## Abstract

As community pharmacies provide essential pharmaceutical and public health services, they should be geographically accessible to populations globally. This study aimed to explore community pharmacy density and associated factors at national and subnational levels in New Zealand (NZ). Publicly available, aggregate data for 2020 were sourced. Community pharmacy density (number of community pharmacies per 10,000 population) was calculated at the national level and at the subnational level using territorial authority (TA) areas. Associations between four TA characteristics (type [cities or districts], island [North or South], older age and deprivation score) and continuous community pharmacy density were assessed via linear regression. There were 1,154 community pharmacies. Numbers of community pharmacies per 10,000 population were 2.27 for NZ, 2.25 and 2.33 for the North and South Islands, respectively, and 2.45 and 1.95 for cities and districts, respectively. The only TA characteristic associated with community pharmacy density was TA type: cities had significantly higher community pharmacy density than districts (adjusted β = 0.58, 95% confidence interval = 0.13–1.02). TAs with lowest-quintile pharmacy densities (≤1.40) were clustered towards the south of the North Island and south-west of the South Island. This study could inform development of policies aimed at providing equitable access to community pharmacies throughout NZ.

## Introduction

Community pharmacies are central to primary healthcare systems in that they provide various pharmaceutical and public health services (McDonald et al. 2021; Schenkelberg et al. 2023). In New Zealand (NZ), for example, all community pharmacies dispense prescribed medications, sell non-prescription medications, and educate patients about medication use, with many also offering professional services such as vaccination, non-prescription supply of the emergency contraceptive pill, blood pressure monitoring, and smoking cessation counselling (McDonald et al. 2021). Access to community pharmacies is essential for all population subgroups, although some subgroups (e.g. older people) may have greater medical need for pharmacy services than others (Grootendorst 2022). Access to health services has been defined with respect to five dimensions: availability, affordability, accommodation, acceptability and (geographical) accessibility (Penchansky and Thomas 1981). Access to health services such as community pharmacies has mainly been measured in relation to availability and affordability; relatively little effort has been invested in measuring other domains of access, including geographical accessibility (Jagadeesan and Wirtz 2021). Geographical accessibility refers to real-world connections between the locations of services and the locations of service users (Penchansky and Thomas 1981).

A commonly used measure of geographical accessibility of community pharmacies is community pharmacy density. Community pharmacy density is typically measured in terms of the number of community pharmacies per 10,000 population (Jagadeesan and Wirtz 2021). The number of community pharmacies per 10,000 population has been determined at national levels for many countries around the world (Norris et al. 2014; Ward et al. 2014; Qato et al. 2017; International Pharmaceutical Federation 2021; Jagadeesan and Wirtz 2021; Berenbrok et al. 2022; Grootendorst 2022; Xiao et al. 2022; Chen et al. 2023). In a study conducted across 77 countries in 2021, the mean national community pharmacy density was 2.75 community pharmacies per 10,000 population, with density ranging from 0.31 in Sudan to 6.84 in Egypt (International Pharmaceutical Federation 2021). Among individual studies that have reported (or provided the data necessary to calculate) community pharmacy density at national levels (Norris et al. 2014; Ward et al. 2014; Qato et al. 2017; Jagadeesan and Wirtz 2021; Berenbrok et al. 2022; Grootendorst 2022; Xiao et al. 2022; Chen et al. 2023), the number of community pharmacies per 10,000 population ranged from 0.61 in South Africa during 2012 (Ward et al. 2014) to 3.48 for Taiwan in 2020 (Chen et al. 2023). Subnational analysis and mapping of the number of community pharmacies per 10,000 population has been conducted in some past studies, thereby permitting the identification of smaller geographic areas where there are insufficient numbers of community pharmacies (Norris et al. 2014; Ward et al. 2014; Emmerick et al. 2015; Qato et al. 2017; Tew et al. 2021; Berenbrok et al. 2022; Grootendorst 2022; Xiao et al. 2022).

Certain population-level characteristics of subnational areas, such as rurality and medical need, may be associated with community pharmacy density. Over time, there has been a general shift towards urbanisation of health services such as community pharmacies (Norris et al. 2014). A US study found that the number of community pharmacies per 10,000 population in the state of Illinois during 2001 was only 0.38 in rural areas yet higher at 1.27 in urban areas (Lin 2004) while a South African study found that the number of community pharmacies per 10,000 population during 2012 was only 0.27 in the most rural province but 0.99 in the most urban province (Ward et al. 2014). As areas with greater medical need require more community pharmacies, the medical need for pharmacy services in a given geographic area is another relevant consideration. Those subnational areas with low community pharmacy density yet high medical need for pharmacy services may be considered under-serviced and, thus, priorities for establishment of new pharmacies. As medical conditions are often treated with medications and the number of medical conditions increases with age and deprivation, the proportion of a population aged 65 years or older (65+ years) and the level of deprivation may be considered demographic markers of medical need for pharmacy services (Grootendorst 2022). In the Canadian context, multiple linear regression analysis showed that the number of community pharmacies per 10,000 population in 2019 was significantly higher in those subnational areas with higher proportions of their populations aged 65+ years and significantly lower in those subnational areas with higher household income (Grootendorst 2022). In a given country, characteristics of subnational areas that are associated with community pharmacy density could also help inform healthcare planning and policy decisions.

In the NZ context, community pharmacy density has been reported at the national level. In an international study set during 2021, NZ had the 48th highest community pharmacy density among 77 countries: 2.19 community pharmacies per 10,000 population (International Pharmaceutical Federation 2021). A separate study (Norris et al. 2014) produced maps of community pharmacy locations and 25 km-radius pharmacy service areas throughout NZ for the years 1954, 1980, and 2010, and provided the data necessary for authors of a global systematic review (Jagadeesan and Wirtz 2021) to calculate community pharmacy density at the national level in NZ. This systematic review reported a community pharmacy density of 2.20 pharmacies per 10,000 population for NZ in 2010 (Jagadeesan and Wirtz 2021). No known studies, however, have reported community pharmacy density and associated factors at the subnational level in NZ.

The present study aimed, firstly, to explore community pharmacy density at national and subnational levels in NZ and, secondly, to determine whether any particular characteristics of NZ’s subnational areas (e.g. older age and deprivation) are associated with community pharmacy density.

## Methods

### Study design

An ecological study was undertaken using aggregate, population-level data.

### Setting

The present study is set during 2020 at the national level in NZ as well as at the subnational, or more specifically territorial authority (TA)-area, level. In NZ, TAs are second-tier, administrative geographic areas that sit below the larger regional councils and above the smaller statistical area (SA) levels. The boundaries of TAs have been defined through consideration of road access and subpopulations of interest. There are 67 TAs: 12 city councils (i.e. councils that are predominately urban with populations ≥50,000) and 53 district councils (i.e. predominately non-urban councils), as well as Auckland Council and Chatham Islands Council (Stats NZ 2023; Parliamentary Council Office 2024). TAs are likely to be suitable for the present study because they account for road access – a key consideration in geographical accessibility of community pharmacies – and permit comparison of community pharmacy density between cities and districts, in a similar way to past studies set beyond NZ (Lin 2004; Ward et al. 2014).

### Data sources

Data on community pharmacy locations, specifically street addresses, across NZ as at 4 April 2020 were sourced from the Ministry of Health and Eagle Technology (2020). Data on NZ’s TA boundaries, usual resident population by TA, and percentage of older residents aged 65+ years by TA as at 30 June 2020 were sourced from Stats NZ (2020, 2022). Deprivation data by SA1 areas (i.e. the smallest of three SA levels below TA areas) for the year 2018, along with correspondences between SA1s and TAs, were sourced from the University of Otago (2025). Four datasets were used: an ArcGIS Pro Portal Item (.pitemx) file containing a point layer of geocoded community pharmacy street addresses, a shapefile (.shp file) containing a polygon layer of TA boundaries, a Microsoft Excel (.xlsx) file containing NZ’s population counts and percentages aged 65+ years by TA, and a Microsoft Excel file containing NZ’s deprivation data by SA1s and correspondences between SA1s and TAs.

### Eligibility criteria

The street addresses of all community pharmacies in NZ as at 4 April 2020 were included in the analysis. Pharmacy street address and population data for all TAs except Chatham Islands Territory were included, in line with a previous NZ mapping study that excluded data for the Chatham Islands (Wiki et al. 2021). Therefore, data for 66 TAs – rather than the full set of 67 TAs – were used in the primary analysis.

### Territorial authority characteristics

TA type was defined as a binary variable with categories of ‘city’ (city councils or Auckland) and ‘district’ (district councils). TA location was defined as a binary variable with categories of ‘North Island’ and ‘South Island’. In line with a past Canadian study (Grootendorst 2022), the extent of medical need for pharmacy services in each TA was assessed using two continuous variables as markers: the percentage of each TA’s population aged 65+ years and the deprivation score for each TA. The deprivation score for each TA was assessed in terms of 2018 New Zealand Index of Deprivation (NZDep) scores, where higher scores denote greater deprivation (University of Otago 2025). Each TA’s deprivation score was determined by calculating the median NZDep score across all SA1 areas in the given TA. Each TA’s median deprivation decile was also calculated.

### Geocoding

In the.pitemx file, NZ’s street addresses had already been geocoded to form a point layer. In order to check this, geocoding was repeated in ArcGIS Pro (Esri, Redlands, CA, USA) via the Esri World Geocoder. As consistency was found (data not shown), the original point layer of geocoded community pharmacy street addresses was used in this study.

### Data management

Data management was undertaken in ArcGIS Pro. The attribute table associated with the polygon layer of TA boundaries was joined to the standalone tables of population data by TA, culminating in an updated polygon layer. This updated polygon layer was then spatially joined to the point layer comprising geocoded community pharmacy street addresses, giving the final dataset for analysis.

### Data analysis

For NZ as a whole, the North and South Islands of NZ separately, cities and districts separately, and each of NZ’s 66 TAs separately, community pharmacy density per 10,000 population was calculated by dividing the count of community pharmacies by the corresponding population count and multiplying by 10,000. Community pharmacy density was treated as a continuous variable, for the purposes of regression analysis, as well as an ordinal variable with quintile categories, for the purposes of data visualisation. Continuous variables for community pharmacy density, the percentage of older residents, and the deprivation score were summarised using the median and interquartile range. Binary variables for the type of TA (city or district) and location (North Island or South Island) were summarised using frequencies and percentages. Pearson’s correlation coefficient (r) was calculated to assess the strength of the correlation between community pharmacy density and each of the percentage of older residents and the deprivation score. As community pharmacy density was found to be normally distributed via histogram inspection (data not shown) and a Shapiro–Wilk test (*p*-value = 0.247), linear regression was used to assess associations between each of the four TA characteristics and community pharmacy density measured on a continuous scale. Unadjusted and adjusted regression coefficients (βs) were calculated in univariable and multivariable linear regression models, respectively, along with corresponding 95% confidence intervals (CIs) and *p*-values. All statistical analysis was undertaken in Stata v15.0 (StataCorp, College Station, TX, USA).

### Data visualisation

**Figure 1.**
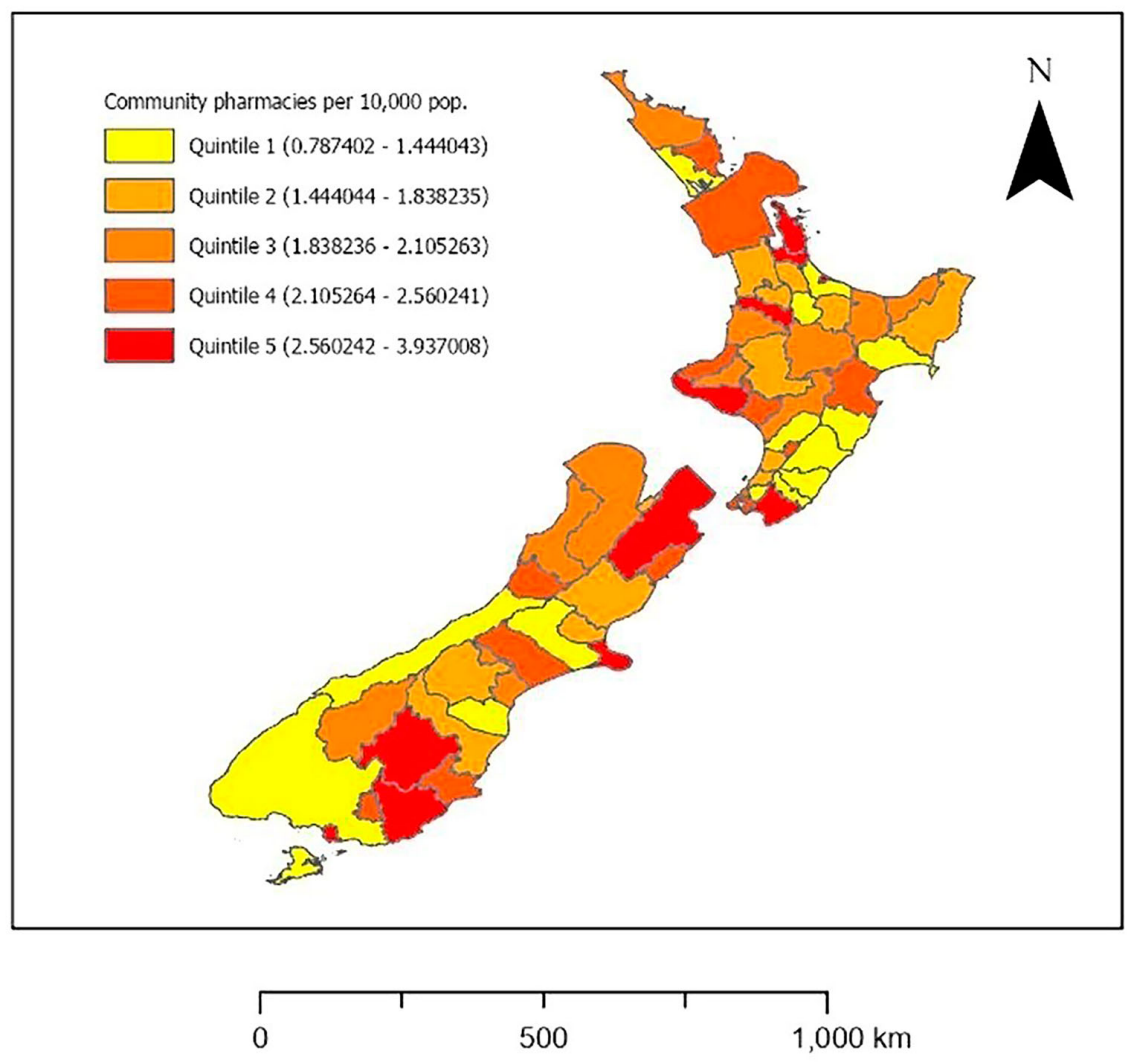
Quintiles of community pharmacy density (number of community pharmacies per 10,000 population [pop.]) in each territorial authority, New Zealand, 2020.

**Figure 2.**
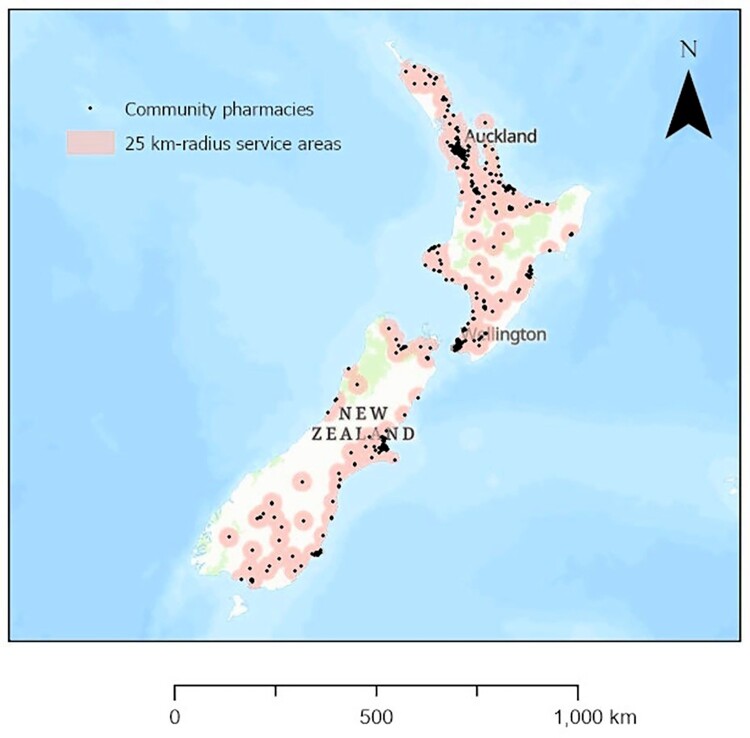
Community pharmacies and 25 km-radius community pharmacy service areas, New Zealand, 2020 (*N* = 1,154 community pharmacies).

A choropleth map showing community pharmacy density quintiles across all TAs was produced (Figure 1). Community pharmacy density by TAs was symbolised as quintiles of the number of community pharmacies per 10,000 population, in line with the symbology approach used in past studies of geographical access to community pharmacies in countries such as the US, Brazil and China (Emmerick et al. 2015; Qato et al. 2017; Berenbrok et al. 2022; Xiao et al. 2022). Additionally, to help further visualise and contextualise community pharmacy density across the North and South Islands of NZ, the point layer of NZ’s community pharmacy locations was mapped with 25 km-radius service areas (Figure 2) obtained via ArcGIS Pro’s ‘Buffer’ and ‘Intersect’ functions. Twenty-five km-radius service areas were used to provide another point of comparison with the past NZ study in this research area (Norris et al. 2014). A Euclidian distance ≤25 km approximates a reasonable distance for customers to travel to a community pharmacy (Norris et al. 2014). All data visualisation was undertaken in ArcGIS Pro 3.1.

### Sensitivity analysis

A sensitivity analysis was undertaken to assess the impact of excluding data for Chatham Islands Territory from the analysis. This involved recalculating community pharmacy density for NZ with Chatham Islands Territory included.

### Ethical considerations

Ethics approval for this study was obtained from the Monash University Human Research Ethics Committee (ID: 46001).

## Results

During 2020, there were 1,154 community pharmacies across NZ’s 66 TAs (excluding Chatham Islands). Overall for NZ, community pharmacy density was 2.27 community pharmacies per 10,000 population. Numbers of community pharmacies per 10,000 population were 2.25 and 2.33 for the North and South Islands, respectively, and 2.45 and 1.95 for cities and districts, respectively (Table 1).

**Table 1.** Number of community pharmacies, resident population, community pharmacy density (number and quintile of community pharmacies per 10,000 population), percentage of population that is older (65+ years), deprivation score and deprivation decile by territorial authority, New Zealand, 2020.

Territorial authority area	Island	% older (65+ years)	Deprivation score^a^	Deprivation decile^a^	Pharmacies	Resident pop.	Pharmacies per 10,000 pop.	
							Number	Quintile
Kawerau District	North	20.9	1,164	10	3	7,620	3.94	5
Thames-Coromandel District	North	32.2	1,001	7	12	32,500	3.69	5
South Taranaki District	North	16.2	1,043	8	10	29,000	3.45	5
Napier City	North	19.9	988	6	20	66,200	3.02	5
Invercargill City	South	17.5	1,011	7	17	57,200	2.97	5
Marlborough District	South	22.2	967	5	15	51,300	2.92	5
Central Otago District	South	22.5	932	3	7	24,300	2.88	5
Tauranga City	North	19.4	971	5	44	153,000	2.88	5
Hauraki District	North	24.4	1,043	8	6	21,500	2.79	5
Otorohanga District	North	15.6	1003	7	3	10,750	2.79	5
Christchurch City	South	15.2	970	5	109	391,600	2.78	5
Clutha District	South	18.1	976	6	5	18,450	2.71	5
South Wairarapa District	North	22.8	960	5	3	11,400	2.63	5
Dunedin City	South	16.6	976	6	34	132,800	2.56	5
New Plymouth District	North	18.3	986	6	22	86,300	2.55	4
Ashburton District	South	18.0	928	5	9	35,700	2.52	4
Auckland	North	12.5	970	5	418	1,714,200	2.44	4
Kaikoura District	South	23.1	964	5	1	4,200	2.38	4
Lower Hutt City	North	13.7	980	6	26	111,800	2.33	4
Wellington City	North	20.1	940	4	50	216,500	2.31	4
Gore District	South	10.6	977	6	3	13,000	2.31	4
Whanganui District	North	21.2	1,045	8	11	47,900	2.30	4
Palmerston North City	North	14.4	992	6	20	90,400	2.21	4
Whangarei District	North	19.4	1,013	7	21	97,800	2.15	4
Grey District	South	19.2	1,011	7	3	14,100	2.13	4
Hastings District	North	16.7	999	6	19	89,400	2.13	4
Whakatane District	North	17.9	1,032	8	8	38,000	2.11	4
Queenstown-Lakes District	South	10.3	911	2	10	47,700	2.10	3
Waitomo District	North	16.4	1,053	8	2	9,680	2.07	3
Buller District	South	26.2	1,064	8	2	9,770	2.05	3
Hamilton City	North	11.8	1,018	7	36	177,400	2.03	3
Stratford District	North	17.7	1,009	7	2	10,000	2.00	3
Porirua City	North	12.2	984	6	12	60,600	1.98	3
Taupo District	North	20.0	992	6	8	40,500	1.98	3
Far North District	North	19.8	1,100	9	14	71,400	1.96	3
Opotiki District	North	17.4	1,192	10	2	10,250	1.95	3
Tasman District	South	21.6	962.5	5	11	57,300	1.92	3
Rangitikei District	North	19.1	1,014	7	3	15,900	1.89	3
Timaru District	South	22.0	976	5	9	48,300	1.86	3
Mackenzie District	South	16.6	929	3	1	5,440	1.84	3
Nelson City	South	19.7	985	6	10	54,900	1.82	2
Rotorua District	North	14.5	1,034	8	14	77,100	1.82	2
Gisborne District	North	15.7	1,061	8	9	51,400	1.75	2
Kapiti Coast District	North	26.1	965	5	10	57,200	1.75	2
Waitaki District	South	22.8	982	6	4	23,700	1.69	2
Horowhenua District	North	24.6	1,053	8	6	35,900	1.67	2
Waikato District	North	13.0	977	6	14	83,800	1.67	2
Matamata-Piako District	North	20.4	1,001	7	6	36,400	1.65	2
Waimakariri District	South	19.9	936	3	10	64,700	1.55	2
Ruapehu District	North	16.8	1,047	8	2	12,950	1.54	2
Waipa District	North	18.2	958	5	9	58,500	1.54	2
Hurunui District	South	21.0	952	4	2	13,450	1.49	2
Masterton District	North	21.6	1,019	7	4	27,700	1.44	2
Western Bay of Plenty District	North	21.7	968	5	8	57,000	1.40	1
Central Hawke’s Bay District	North	20.4	990	6	2	15,400	1.30	1
Upper Hutt City	North	12.0	972	5	6	46,800	1.28	1
Selwyn District	South	14.9	903	2	9	70,300	1.28	1
Southland District	South	15.8	957	5	4	32,600	1.23	1
Waimate District	South	23.2	982	6	1	8,270	1.21	1
South Waikato District	North	16.7	1,082	9	3	25,400	1.18	1
Westland District	South	17.9	995	6	1	8,910	1.12	1
Wairoa District	North	19.1	1,159	10	1	8,940	1.12	1
Tararua District	North	19.1	1,028	7	2	18,800	1.06	1
Carterton District	North	23.7	959	5	1	9,890	1.01	1
Manawatu District	North	18.3	979	6	3	32,600	0.92	1
Kaipara District	North	23.2	1,028	7	2	25,400	0.79	1
Cities (13 TAs)^b^	Both	13.6	980	5	802	3,273,400	2.45	N/A
Districts (53 TAs)	Both	19.0	995	6	352	1,815,770	1.94	N/A
North Island (43 TAs)	North	15.0	1,003	5	877	3,901,180	2.25	N/A
South Island (23 TAs)	South	17.3	970	7	277	1,187,990	2.33	N/A
New Zealand (All 66 TAs)	Both	15.6	987	5	1,154	5,089,170	2.27	N/A

Pop. – population, TA – territorial authority, N/A – not applicable.

^a^ Median 2018 New Zealand Index of Deprivation (NZDep) score/decile for each TA, calculated from the NZDep scores/deciles for all SA1 areas in a given TA.

^b^ ‘Cities’ include the 12 city TAs plus Auckland.

Figure 1 shows quintiles of community pharmacy density for each of NZ’s 66 TAs (43 TAs on the North Island and 23 TAs on the South Island), while Table 1 shows the exact community pharmacy density for each TA (in descending order) alongside the percentage of older residents, deprivation score, and deprivation decile. Some adjacent TAs had highest-quintile (quintile 5) community pharmacy density: two on the North Island (Thames-Coromandel District and Hauraki District) and two on the South Island (Central Otago District and Clutha District). NZ’s most populous TA, Auckland, had a community pharmacy density in the second-highest quintile yet relatively few older residents (12.5%) while NZ’s next most populous TA, Christchurch City, had a community pharmacy density in the highest quintile despite having a proportion of older residents (15.2%) slightly below that of NZ overall (15.6%). Figure 2 reveals marked clustering of individual pharmacies in Auckland and, to a lesser extent, Christchurch City. Clusters of lowest-quintile (quintile 1) community pharmacy density are evident for six smaller TAs located towards the south of the North Island (i.e. Upper Hut City, Carterton District, Masterton District, Tararua District, Central Hawke’s Bay District and Manawatu District) as well as two geographically larger TAs located at the south-west of the South Island (i.e. Southland District and Westland District). Among the 13 TAs with lowest-quintile community pharmacy density, there were 12 districts and only 1 city as well as 11 TAs whose proportions of older residents exceeded the national proportion of 15.6% (Table 1).

When adjusted for the percentage of older residents, deprivation score and whether each TA was located on the North Island or the South Island, cities had statistically significantly higher community pharmacy density than districts (adjusted β = 0.58; 95% CI = [0.13, 1.02]) (Table 2). When adjusted for one another and whether each TA was a city or a district, the percentage of older residents, deprivation score and the particular island of NZ were unrelated to community pharmacy density (Table 2). The r for the relationship between TAs’ percentage of older residents and community pharmacy density was 0.081, while the r for the relationship between TAs’ deprivation score and community pharmacy density was 0.053.

**Table 2.** Associations between territorial authority area characteristics and increasing community pharmacy density (*N* = 66 territorial authorities).

Characteristic	*n* (col. %)^a^	Univariable effect		Multivariable effect	
		Unadjusted β (95% CI)	*p*-value	Adjusted β (95% CI)	*p*-value
% older (65+ years)	*M* (IQR) = 19.1 (5.2)	0.013 (−0.028, 0.054)	0.520	0.032 (−0.011, 0.075)	0.143
Deprivation score	M (IQR) = 987 (60)	0.001 (−0.002, 0.004)	0.670	0.002 (−0.002, 0.005)	0.305
City					
No (District)	53 (80.3%)	0			
Yes (City)	13 (19.7%)	0.408 (0.002, 0.813)	0.049^b^	0.577 (0.133, 1.020)	0.012*
Island					
North	43 (65.2%)	0			
South	23 (34.8%)	0.046 (−0.302, 0.395)	0.791	0.149 (−0.231, 0.530)	0.435

n – frequency/numerator, col. – column, β – linear regression coefficient, CI – confidence interval, M – median, IQR – interquartile range.

^a^ Unless otherwise stated.

^b^ Statistically significant at the 5% level.

Figure 2 also shows the locations of community pharmacies and 25 km-radius community pharmacy service areas for NZ in 2020. Visual inspection revealed that, compared with the North Island, a lower proportion of the South Island’s land was covered by community pharmacy service areas in 2020.

In the sensitivity analysis, the following additional data for Chatham Islands Territory were included: 0 community pharmacies and a resident population of 750. NZ’s national pharmacy density of 2.27 pharmacies per 10,000 population remained unchanged when data for Chatham Islands Territory were included.

## Discussion

The present ecological study found that, at the national level in NZ during 2020, there were 1,154 community pharmacies overall and a density of 2.27 community pharmacies per 10,000 population. NZ’s community pharmacy density is 0.48 lower than the global mean of 2.75 community pharmacies per 10,000 population (International Pharmaceutical Federation 2021). This finding is somewhat at odds with the fact that NZ has a relatively high level of human development, as indicated by a human development index of 0.937 (United Nations Development Program 2022). NZ’s community pharmacy density in the present study is, however, similar to that reported in previously published studies: 2.20 and 2.19 community pharmacies per 10,000 population during 2010 and 2021, respectively (Norris et al. 2014; International Pharmaceutical Federation 2021; Jagadeesan and Wirtz 2021). As NZ has relatively poor public transport services across urban and non-urban areas (Muhammad and Pearce 2015), the country’s community pharmacy density may need to be increased beyond the global mean of 2.75 pharmacies per 10,000 population to make pharmacy services more available and accessible to people nationally.

The present study is the first known investigation into community pharmacy density at the subnational level in NZ. While the number of community pharmacies per 10,000 population was similar between the entire North Island (2.25) and the entire South Island (2.33), there was considerable variation in community pharmacy density among the 66 TAs located across these two islands. Those TAs with the lowest community pharmacy densities were clustered on the south-west of the South Island and towards the south of the North Island, geographically distant to each island’s largest city where pharmacies were mostly concentrated (Auckland and Christchurch City). On the South Island, the lowest-quintile community pharmacy density values for Southland District and Westland District could reflect the fact that these geographically large TAs have among the lowest population densities of all NZ TAs: 1.05 people per km^2^ and 0.75 people per km^2^, respectively (author calculations using TA area data accessed online [Stats NZ 2020] and population data in Table 1). It is, nonetheless, inequitable and unjust for these less densely populated TAs to have so few community pharmacies per 10,000 population (Hayes et al. 2025). While there are no previously published data on NZ’s community pharmacy density by subnational areas, the observed tendency for geographic areas with lowest-quintile pharmacy densities to be clustered rather than evenly distributed is supported by US studies (Qato et al. 2017; Berenbrok et al. 2022). New Zealanders residing in contiguous areas of low community pharmacy density, or pharmacy deserts (Jagadeesan and Wirtz 2021), may require new local community pharmacies to be established and/or greater access to other health services (e.g. mail-order pharmacies and local centres/clinics that provide vaccination services).

In the present NZ study, community pharmacy density was found to be statistically significantly higher in cities than districts. This finding is partially supported by the results of past studies. Community pharmacy densities were 2.45 and 1.94 for NZ’s cities and districts, respectively, in 2020, compared with 1.27 and 0.38 for Illinois’s urban and rural areas, respectively, in 2001 (Lin 2004), and 0.99 and 0.27 for South Africa’s most urban and most rural provinces, respectively, in 2012 (Ward et al. 2014). A possible explanation for the viability of so many community pharmacies in cities is the selling of front-of-shop products to city dwellers of all ages and levels of medical need – a potential avenue for future research (Pharmaceutical Society of New Zealand Incorporated 2017). The significantly higher community pharmacy density in NZ’s cities is consistent with the fact that, in 2020, more pharmacists (community or non-community) per 10,000 population were employed in NZ’s cities than in rural NZ (Pharmacy Council 2020). This discrepancy may reflect a pharmacy workforce issue. While the type and extent of rural pharmacy workforce issues in the NZ context are unclear and require future investigation, a likely explanation is a dearth of rural training initiatives for community pharmacists in NZ (O’Sullivan and Worley 2020; Walker et al. 2022).

The observed lack of statistically significant associations between community pharmacy density and each of the percentage of older residents and deprivation score are supported by very low r values – *r* values that denote very weak correlations. These unexpected results contrast with a Canadian study that reported a statistically significant positive association between subnational areas’ percentage of older residents and community pharmacy density, along with a statistically significant negative association between subnational areas’ household income and community pharmacy density (Grootendorst 2022). As the percentage of older residents and deprivation score were unrelated to community pharmacy density in the present NZ study, the relatively high medical needs of certain NZ TAs may be unmet. Examples of such under-serviced TAs are Kaipara District on the North Island and Waimate District on the South Island, where 23% of each population was aged 65+ years but numbers of community pharmacies per 10,000 population were only 0.79 and 1.21, respectively (Table 1). When considered alongside the significantly higher community pharmacy density in cities than in districts, the lack of significant associations between markers of medical need and community pharmacy density may reflect human decisions to preferentially invest in the health facilities of urban areas without giving due consideration to the extent of deprivation and older age in geographic areas (Hayes et al. 2025). In other words, community pharmacy density in NZ may be driven by the extent of urbanisation at the expense of medical needs.

The higher community pharmacy service area coverage in the North Island than South Island during 2020 is consistent with a previously published map of NZ’s community pharmacy service areas in 2010 (Norris et al. 2014). This difference between islands is expected due to their varied population densities. In 2020, the South Island had a lower population density of 7.9 people per km^2^ while the North Island had a higher population density of 34.3 people per km^2^ (author calculations based on area data accessed online [Stats NZ 2000] and population data in Table 1).

The present study’s results should be interpreted in light of seven limitations. Firstly, as data on community pharmacy addresses were dated 4 April 2020 and the data on population counts by TA were dated 30 June 2020, there may have been a slight discrepancy in the accuracy of numerators and denominators. Secondly, the data are five years old at the time of writing; community pharmacy density at national and subnational levels in NZ likely changed between 2020 and 2025. In NZ during the COVID-19 pandemic, community pharmacies remained open, experienced increased workloads, and played a central role in COVID-19 vaccination (Officer et al. 2024). While some of the 1,154 NZ community pharmacies that were open in 2020 may have subsequently closed as new ones opened, potentially precipitating a change in the net number of community pharmacies, there are still >1,000 NZ community pharmacies at the time of writing (Health New Zealand 2025). Thirdly, as with any subnational analysis of community pharmacy density, TAs with smaller population sizes are by definition more sensitive to slight changes in the number of community pharmacies. Fourthly, the percentage of each TAs population aged 65+ years and deprivation score are imperfect markers of medical need for pharmacy services. For instance, there are likely some people aged less than 65 years who have greater medical need than some people aged 65+ years. A better marker of medical need for pharmacy services in a given TA would be the number of chronic medical conditions among the population. Fifthly, the lack of statistically significant associations between community pharmacy density and each of percentage of older residents, deprivation score and island could be explained by the somewhat low sample size of 66 that is inherent to TA-level analysis, although descriptive statistics do support the absence of associations. Sixthly, the observed associations between TA characteristics and community pharmacy density may have been confounded by unmeasured factors associated with both the independent and dependent variables (e.g. health status of the population). Lastly, community pharmacy density was not considered alongside other types of geographical accessibility, such as driving time (Sharareh et al. 2024) and walking distance (Padeiro 2018), or other elements of access (Penchansky and Thomas 1981), such as accommodations designed to make community pharmacies more accessible (e.g. longer operating hours, acceptance of e-prescriptions and home delivery of medications) (Qato et al. 2017). Driving time is a particularly important consideration for those who live far from their closest or preferred community pharmacy, particularly geographically isolated non-urban residents (Sharareh et al. 2024). The chosen subnational areas in the present study did, however, account for road access within each TA (Stats NZ 2023).

## Conclusions

This study is novel in that it is the first to report community pharmacy density and markers of medical need for pharmacy services at the subnational level in NZ. While NZ’s nationwide community pharmacy density in 2020 was close to the global mean at 2.27 community pharmacies per 10,000 population, density varied across TAs and was significantly greater in cities than districts. Future studies in this research area should track NZ’s subnational community pharmacy density and population change longitudinally at different time points, measure community pharmacy density alongside other measures of access, and measure morbidity as a marker of medical need. From an equity perspective, future studies should consider NZ’s subnational community pharmacy density by ethnic groups such as Māori and Pacific peoples – data that were unavailable in the subnational population dataset used in the present study (Stats NZ 2022). Future studies could also examine the impact of poor geographical accessibility of community pharmacies on health outcomes (e.g. quality of life and mortality). Despite this scope for further research, the present study could still inform the development of policies and legislation aimed at providing equitable access to community pharmacies across all areas of NZ. For instance, the present study’s finding that community pharmacy density has been inequitably driven by urbanisation rather than markers of medical need could inform rural pharmacist training initiatives as well as community pharmacy ownership considerations in the Ministry of Health’s Medical Products Bill – a bill that is under development at the time of writing (Pharmaceutical Society of New Zealand Incorporated 2017; Ministry of Health 2025).
